# Development and evaluation of planar GSM antenna integra-tion into the housing of a navigation seal for transport and logistics

**DOI:** 10.1038/s41598-026-53012-6

**Published:** 2026-05-26

**Authors:** Altay Aitmagambetov, Sabyrzhan Zhumagali, Aigul Kulakayeva, Anatoly Samsonenko, Mikhail Saiganov

**Affiliations:** 1 International Information Technology University, Almaty, Kazakhstan; 2 Institute of Space Technique and Technology, Almaty, Kazakhstan

**Keywords:** Engineering, Materials science

## Abstract

The article presents the results of a comparative study of 28 types of commercial flat GSM antennas and a self-developed antenna “Zmeyka” fabricated on a 1 mm FR-4 substrate operating in the GSM 900 band (Uplink 890 MHz, Downlink 960 MHz) for integration into the housing of a navigation seal intended for real transportation conditions. Particular attention is paid to the influence of housing materials, layout constraints, and the electromagnetic environment on impedance matching parameters and signal reception quality. The study was conducted in several stages. Laboratory measurements in an anechoic chamber using a Keysight FieldFox N9915A analyzer showed that in the reference housing made of 2 mm ABS plastic, the “Zmeyka” antenna achieved an average VSWR of 1.2, whereas several commercial samples exhibited VSWR values exceeding 6. Subsequently, selected antennas were tested in field conditions using a proprietary software tool “GSM Antenna Tester” to record CSQ/RSSI parameters. This approach enabled a comparative analysis of antenna performance not only under controlled laboratory conditions but also in scenarios close to real operating environments. Experimental results demonstrated that the integration of antennas into a housing made of reinforced polyamide PA12+GF (*\varepsilon = 3.5*–4.5) with 3.5 mm wall thickness and dense internal layout causes a downward shift of the resonance frequency by approximately 50 MHz, as well as an increase in VSWR up to 3.5 and a reduction of the transmission coefficient in the main lobe by 6–7 dB. Against this background, the self-developed “Zmeyka” antenna showed the highest resistance to layout and environmental effects, maintaining acceptable impedance matching and stable signal reception. Field tests using the “GSM Antenna Tester” software recorded a decrease in the signal level of commercial antennas to the “Weak” category (CSQ 7–11, RSSI from *-99* to *-91* dBm), while the adaptive design of the “Zmeyka” antenna compensated for environmental effects. The obtained results confirm that the design of navigation seals with integrated GSM communication cannot rely solely on the nominal antenna specifications provided by manufacturers. The electromagnetic environment inside the housing, material properties, and layout features have a significantly stronger impact on antenna performance than is typically assumed. It has been demonstrated that the possibility of adjusting the topology of the radiating elements in the “Zmeyka” antenna makes it possible to reduce insertion losses from 6–7 dB to an acceptable level of 1–2 dB, thereby ensuring stable data transmission under challenging transportation operating conditions.

## Introduction

In recent years, particular attention has been paid in the Republic of Kazakhstan to improving the efficiency and safety of cargo transportation, which is associated with the country’s important strategic position within the regional and international transport corridors. Given the growth in freight volumes, stricter requirements for cargo traceability, and increased control over logistics chains, electronic navigation seals have become increasingly relevant. These devices enable the remote real-time monitoring of the location and condition of cargo. Such electronic navigation seals must not only ensure cargo security but also comply with the strict technical and operational requirements established by the Decision of the Council of the Eurasian Economic Commission dated July 4, 2023, No.$\tilde{7}5$^1^. The key requirements include the presence of a replaceable reusable sealing element, automatic integrity monitoring, a unique identification number, the capability for remote control via a national telecommunications operator, a high degree of enclosure protection (IP67 and above), a wide operating temperature range (from – 40 to +70 °C), and ensuring long autonomous operation for at least 30–45 days under various data transmission modes. Currently, electronic navigation seals are becoming increasingly widespread across various modes of freight transportation, including container, road, rail, and air transport. They ensure the monitoring of shipment integrity, geographic coordinates, and motion parameters at all stages of transportation^2^. Freight transportation forms the backbone of global trade, accounting for approximately 80 The deployment of digital containers equipped with sensors and communication modules enables the online monitoring of location, cargo condition, and environmental parameters. This reduces the risk of loss and damage while providing data for transport optimization^3^. Under stricter customs procedures and multilevel border inspections, electronic navigation seals are an important element of a multilayered security system, contributing to improved cargo safety and logistics efficiency^3^. The modern market offers a variety of solutions, among which RFID is widely recognized as a convenient means of cargo identification and control^4^. At the Cikarang Dry Port (Indonesia), active RFID seals with geofencing (iSpot iSensor, iSpot iScout Mini, and iSpot In Vehicle Management) within the ECTS system have demonstrated their effectiveness in industrial logistics^5^. However, dependence on RFID infrastructure and the inability to maintain communication under weak GSM signal conditions limit their applicability in the Eurasian Economic Union (EAEU) environment. In China, more versatile technological solutions are available, including multifunctional navigation seals with GPS/GSM interfaces that support BLE and NFC for interaction with smartphones and remote control of locking mechanisms through mobile applications. These devices have been applied in industries related to the transportation of food products, petrochemicals, and gas products^6^. However, despite their high level of technological sophistication, these solutions do not provide a comprehensive assessment of energy efficiency and communication robustness under prolonged autonomous operation conditions. In several developing countries, such as Bangladesh, Internet of Things (IoT)-based solutions are being actively deployed, combining GPS, GSM/GPRS technologies, and microcontrollers for remote vehicle monitoring and control. In such systems, users can track vehicle movement in real time and perform remote locking via a mobile application, making these solutions particularly effective in combating theft and violations of logistics procedures^7,8^. One of the key development directions for electronic navigation seals (ENS) is ensuring information security and robust communication under various operating conditions. In^9^, a hybrid cryptographic mechanism that combines symmetric and asymmetric encryption and employs identity-based encryption (IBE) methods is proposed. This approach is particularly relevant for devices with limited computational capabilities. However, despite the importance of such solutions, the primary focus is on security protocols, while the physical and radio-frequency aspects of navigation seal design, which are critical for achieving energy efficiency and stable communication, remain insufficiently addressed. In several studies, the focus has shifted toward high-frequency communication channels in enclosed metallic environments. For example, study^10^ investigates a navigation seal operating at 60 GHz, with emphasis on the RDS parameter and influence of enclosure geometry. However, this frequency is not suitable for use in the EAEU context, where the GSM standard frequency band is used. Similar conclusions are supported by studies^11^, which present an intra-cluster ray-tracing model (RT-ICM) for mmWave channels, and^12^, where resonant modes and path losses inside metallic enclosures are analyzed for the THz band. Study^13^ examined antenna-in-package (AiP) technology for frequencies up to 300 GHz, intended for automotive radar applications, but did not meet the requirements for ENS in terms of operating frequency range, dimensions, and energy efficiency. Study^14^ provides useful insights into protection against external electromagnetic interference in terms of the shielding effectiveness and enclosure quality factors. However, it does not address integration with navigation and data transmission modules, which limits its practical applicability to tasks related to ENS.Similarly, studies^15,16^, and^17^ focus on GPS- and GSM-based tracking systems, emphasizing object localization via mobile applications and Google Maps; however, they do not consider sealed enclosures, vibration loads, and constrained power supply conditions that are characteristic of ENS. Intelligent vessel guidance systems^18^ and studies of Arctic routes^19^ highlight the importance of adapting technologies to extreme conditions, including temperature, infrastructure, and navigation factors. These studies emphasize the need to develop robust and energy-efficient solutions for complex logistics scenarios similar to cross-border transportation within the EAEU. In turn^20^, points out the shortcomings of existing multimodal logistics methods with respect to intelligent solutions such as electronic navigation seals, thereby confirming the relevance of their development within integrated logistics systems. The advantages of implementing disposable RFID seals in maritime transportation have been demonstrated to improve cargo traceability^21^. However, these solutions do not provide bidirectional communication or long-term autonomous operation. Modern data transmission technologies based on A-GPS and 4G/LTE significantly reduce the time to the first fix (TTFF), especially in dense urban environments^22^. The implementation of these technologies on platforms such as Raspberry Pi with a RESTful API demonstrates their potential for adaptation to ENS, providing high positioning accuracy and operation in shielded environments. An important direction for future research is institutional integration. The Council of the Eurasian Economic Commission has adopted a decision to implement a Unified System for Goods Traceability in the EAEU countries, which requires the development of compatible, reliable, and regulatorily harmonized technical solutions^23^. In this context, electronic navigation seals are considered a key element of the infrastructure of the EAEU digital transport corridors, including the China–Russia and Europe–Western China routes and the North–South corridor linking Europe, the South Caucasus, Central Asia, and China^24^. In the future, algorithmic approaches to enhance communication robustness^25^ and the application of modern networking solutions to increase data throughput and transmission reliability should be considered^26^. Despite the wide range of commercial solutions available,Kazakhstan still lacks a domestically produced navigation seal that fully complies with the requirements of^1^ and is adaptedto harsh climatic and geographical conditions. Currently, the market is dominated by high-cost imported devices that do not adequately account for the extreme temperatures of northern regions or low GSM signal levels in remote areas. Simultaneously,GSM (2G) technology provides coverage over more than 95Integrating a GSM antenna into the limited space of a navigationseal enclosure poses a significant challenge, which is particularly evident in the changes in the VSWR, shifts in the resonantfrequencies, and increased losses when using plastics such as ABS, PETG, and PA12+GF composites, as demonstrated inthis study. It is necessary to ensure enclosure sealing (IP67 standard), resistance to vibration and temperature loads, andaccount for the close proximity of metallic components and batteries. Practical tests have shown that even minor errors inantenna selection or placement can lead to increased reflected signals (higher VSWR), greater power consumption of the radiomodule, and reduced overall device reliability. The literature review shows that, despite significant progress in the design ofnavigation seals, there is still a lack of systematic, experimentally validated approaches within the engineering and scientificcommunities for selecting and verifying GSM antennas for compact autonomous devices that consider complex operatingconditions. Most existing studies are limited to laboratory experiments or theoretical analyses, often neglecting the specificsof sealed plastic enclosures and real climatic, electromagnetic, and mechanical impacts. In practice, comparative analyses ofcommercial and in-house antennas are rarely conducted under conditions close to real-world operations. The present studyaims to address this existing gap and represents the first systematic attempt in the region to carry out step-by-step development,comprehensive experimental verification, and comparative analysis of a navigation seal prototype in full compliance withthe current requirements^1^. In this study, a multilevel engineering methodology for antenna solution selection was proposedand validated. It includes stages of laboratory and field testing, as well as modeling of extreme operating scenarios that aretypical for navigation seals. A clear and reproducible criterion for antenna comparison was developed, enabling the objectiveselection of solutions for subsequent implementation in industrial production. The obtained results make it possible to developproprietary navigation seals that fully meet the specific requirements of the Republic of Kazakhstan and consider all regionalclimatic, geographical, and infrastructural features. This creates a solid foundation for further technological development in thesector and enhances the competitiveness of national transport and logistics systems in the region.

## Materials and Methods

Within the framework of this study, a comprehensive evaluation of the antenna and GSM radio module of an electronic navigation seal was conducted, the design and operation of which fully comply with the requirements of^1^. To develop the proprietary enclosure of the navigation seal prototype, SolidWorks software was used, enabling a detailed design of the proposed navigation seal while considering therequirements for structural strength, mechanical compatibility of modules, and overall dimensions. The aim of this study was to conduct a comprehensive study and comparative analysis of planar GSM antennas manufactured by Shenzhen Kexin Wireless Technology Co., Ltd. (China) to select the most suitable models for use in a domestically developed electronic navigation seal, considering the operating conditions, dimensional constraints, and energy efficiency. Thus, the aim of this study was not only to compare commercial antenna samples based on their intrinsic radio-frequency characteristics, but also to validate a comprehensive approach to their selection, which includes: - Consideration of enclosure design constraints and installation environment - analysis of electromagnetic matching through the VSWR parameter. - Experimental evaluation of signal reception quality using CSQ/RSSI indicators when integrated with a GSM module. - Experimental assessment of antenna radiation patterns.

**Fig. 1:**
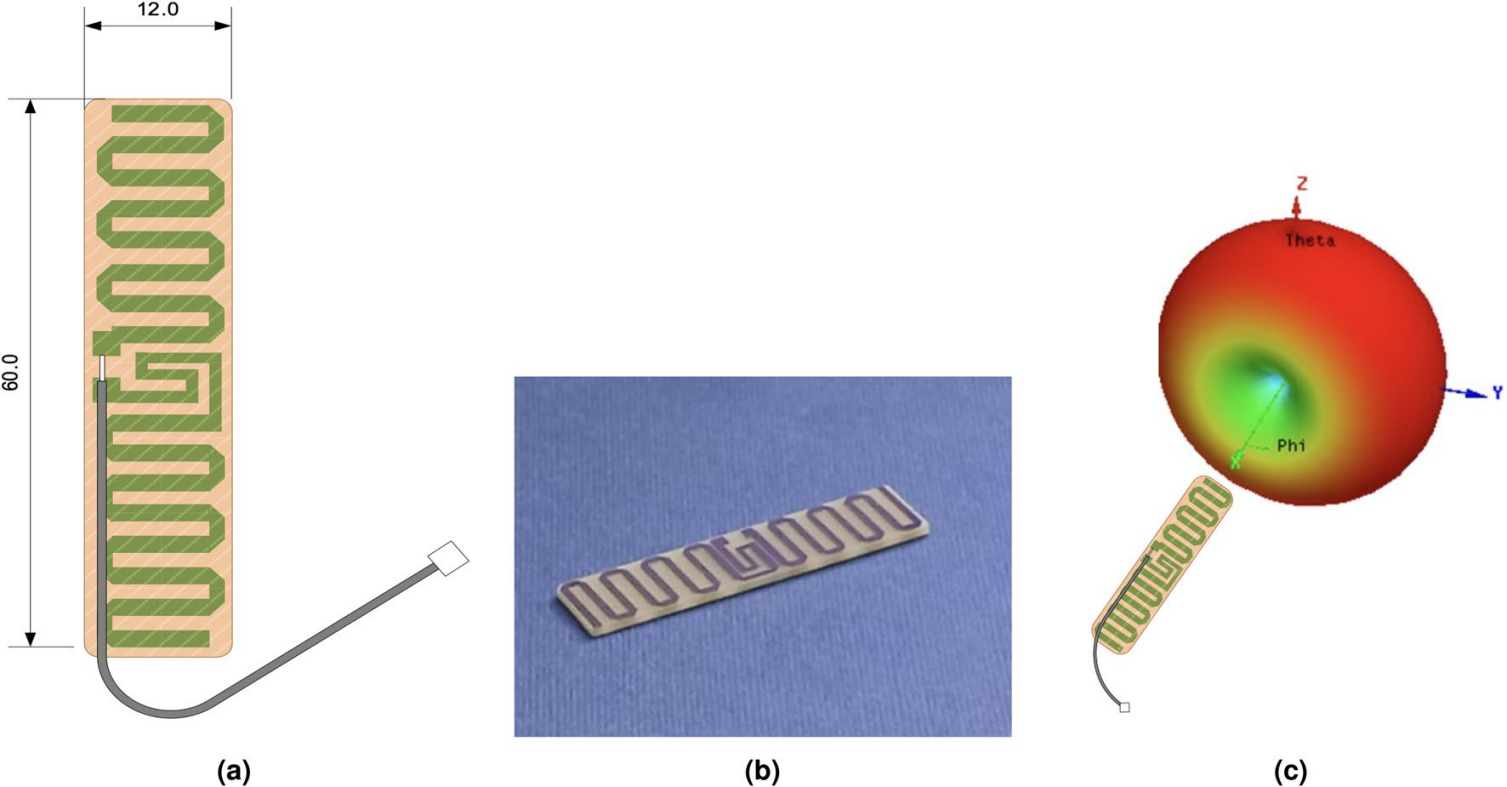
Appearance and drawing of the reference “Snake” antenna: (**a**) schematic diagram; (**b**) photograph of the prototype of the developed antenna; (**c**) radiation pattern.

Within the scope of this study, 28 types of planar commercial GSM antennas from this manufacturer were selected as test objects, each assigned an individual identifier (No. 1–28). For comparative evaluation, the in-house “Snake” antenna developed at LLP “Institute of space technique and technology” on a fiberglass substrate was used as a reference in terms of VSWR in the GSM 900 MHz band (Fig. 1)^27^.

**Fig. 2:**
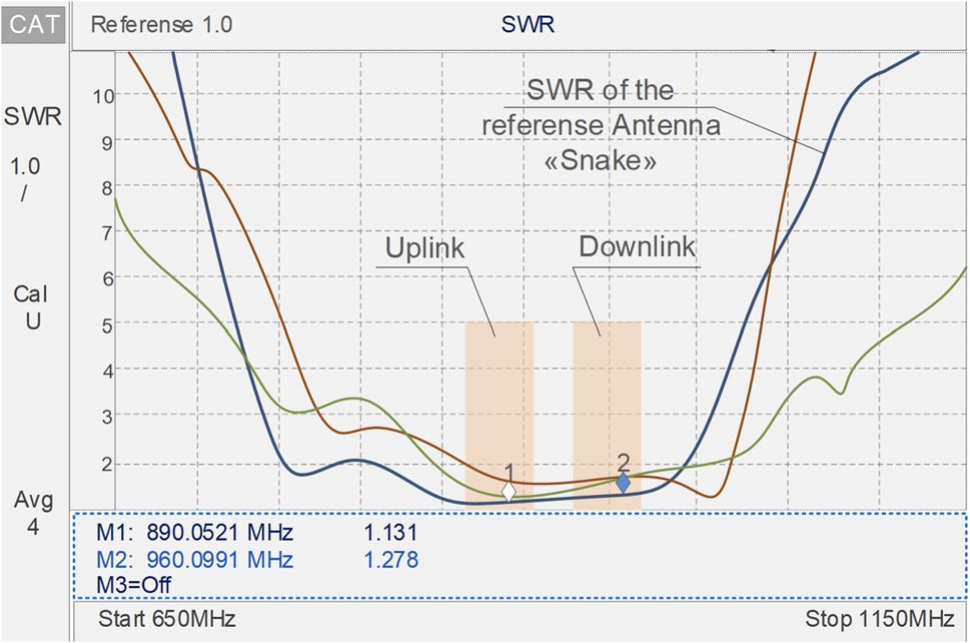
VSWR matching characteristics of the antennas with the best performance: blue trace—VSWR of the “Snake” antenna, green trace—VSWR of antenna No. 1, brown trace—VSWR of antenna No. 25.

This antenna demonstrated the best VSWR performance within the specified frequency range and met the dimensional constraints required for integration into the sealed plastic enclosure of a navigation seal. Therefore, in the first stage, the VSWR characteristics of the investigated antennas were compared with those of the “Snake” antenna, which was used exclusively as a reference (control) for comparative evaluation and was not initially considered for integration into the enclosure of the navigation seal under development. The testing methodology included several stages. Stage 1. Laboratory measurements of the VSWR of commercial antennas on a specialized test bench in an anechoically shielded chamber (ASC). Stage 2. Field measurements of reception quality in the GSM-900 network using the SIM868 module integrated into the navigation seal, with recording of the CSQ parameter considering the RSSI level (dBm) in accordance with 3GPP TS 27.007. Stage 3. Measurement of radiation patterns of several selected antennas housed in enclosures made of different plastics in an anechoic chamber. Commercial antennas were procured; however, the navigation seal enclosure prototypes had not yet been 3D-printed, and some assemblies and components were not sufficiently ready for full system integration. To avoid delays, an approximate plastic enclosure made of 2 mm thick ABS material was used for the VSWR (SWR) measurements. Consequently, a mock-up of the navigation seal plastic enclosure with a relatively spacious internal layout was created. To ensure that the experimental conditions were as close as possible to the real operating conditions, each antenna was fixed to the wall of the plastic enclosure and positioned at a distance of 1 cm from a metal plate intended to simulate internal metallic components. This approach was used to account for the influence of the plastic material on the antenna tuning frequency and impedance matching with the GSM module, as well as to analyze the impact of nearby internal metallic elements, electronic board, power battery, and wiring on antenna characteristics. The meander-type “Snake” antenna designed at that time on a 1 mm thick fiberglass substrate was successfully adapted and impedance-matched to 50 *Ω* over the GSM 900 uplink and downlink frequency bands. For a visual illustration of the frequency characteristics of the antennas that demonstrated the best performance, Fig. 2 presents the VSWR versus frequency curves for antennas No. 1 and No. 25, as well as for the in-house developed “Snake” antenna. Two reference points are marked on each graph: Marker 1 corresponds to the uplink frequency (890 MHz), and Marker 2 corresponds to the downlink frequency (960 MHz).

**Fig. 3:**
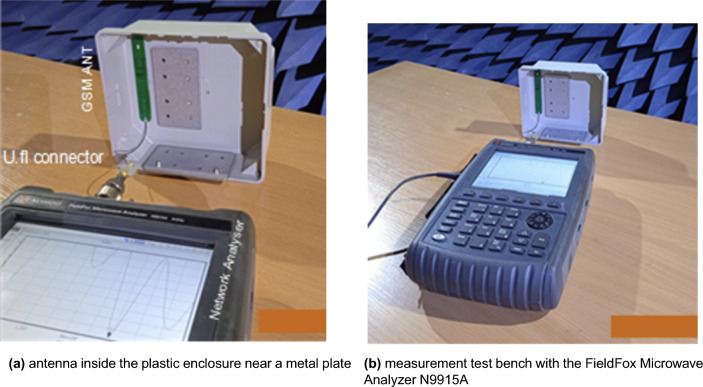
Conditions for VSWR measurements of commercial antennas on the enclosure mock-up.

Figure 3 shows the antenna placement conditions of the measurement test bench and an external view of the laboratory setup. All measurements were performed on the same test bench to ensure the comparability of the results.

Laboratory measurements were conducted in a shielded anechoic chamber of the «Institute of space technique and technology» using a Keysight FieldFox N9915A vector network analyzer at frequencies of 890 MHz (uplink) and 960 MHz (downlink), which correspond to the GSM 900 operating band. For each antenna, the average VSWR value was calculated, after which the result was classified according to the following scale: values below 2 were considered excellent; 2–3 were classified as good; 3–5 were defined as satisfactory; 5–6 were regarded as low quality; and values above 6 were deemed unacceptable for use. It should also be noted that the wooden table on which the setup was placed had a negligible influence on the VSWR (1–2 To objectively assess the effectiveness of antenna impedance matching, this study employed the widely accepted engineering relationship between the VSWR and the fraction of reflected power returned to the transmission line. The fraction of reflected power was calculated using the following equation:

$$\frac{P_{\textrm{refl}}}{P_{\textrm{inc}}} = |\Gamma |^{2} = \left| \frac{Z_{\textrm{ant}} - Z_{c}}{Z_{\textrm{ant}} + Z_{c}}\right| ^{2} = \left| \frac{\textrm{VSWR}-1}{\textrm{VSWR}+1}\right| ^{2}.$$

where $\Gamma = |\Gamma |e^{j\gamma }$ is the voltage reflection coefficient at the antenna input terminals, VSWR is the voltage standing wave ratio at the antenna input terminals, and $Z_c$ is the characteristic impedance of the transmission line. Equation enables the quantitative determination of the effectiveness of the antenna matching to the signal transmission line. Consequently, the lower the VSWR value, the smaller the reflection losses and the higher the efficiency of the antenna system^28^. Table 1 presents the results of the VSWR (SWR) measurements for the in-house developed “Snake” antenna and 28 commercial antennas, mainly based on a polyamide substrate, tested on the enclosure mock-up.

**Table 1:** Results of VSWR measurements of GSM antennas in an ABS plastic enclosure.

Antenna no.	SWR in an ABS enclosure with a thickness of 2 mm		
	Uplink 890 MHz	Downlink 960 MHz	Average SWR
“Snake”	1.13	1.27	1.20
1	1.14	1.55	1.35
2	1.70	1.68	1.69
3	2.51	2.99	2.75
4	2.10	4.37	3.23
5	3.11	1.96	2.53
6	1.66	2.00	1.83
7	2.29	2.32	2.31
8	4.90	5.91	5.41
9	2.35	1.55	1.95
10	10.83	9.00	9.91
11	2.49	5.78	4.14
12	1.32	2.94	2.13
13	1.68	1.74	1.71
14	5.02	6.17	5.60
15	7.95	9.12	8.54
16	2.46	3.20	2.83
17	1.34	1.48	1.41
18	1.40	1.38	1.94
19	1.54	2.62	2.08
20	2.00	2.33	2.17
21	3.62	5.36	4.49
22	3.64	3.33	3.49
23	3.22	3.85	3.54
24	2.51	2.70	2.60
25	1.63	1.52	1.58
26	2.22	2.86	2.54
27	1.65	1.65	1.65
28	6.78	5.28	6.03

**Fig. 4:**
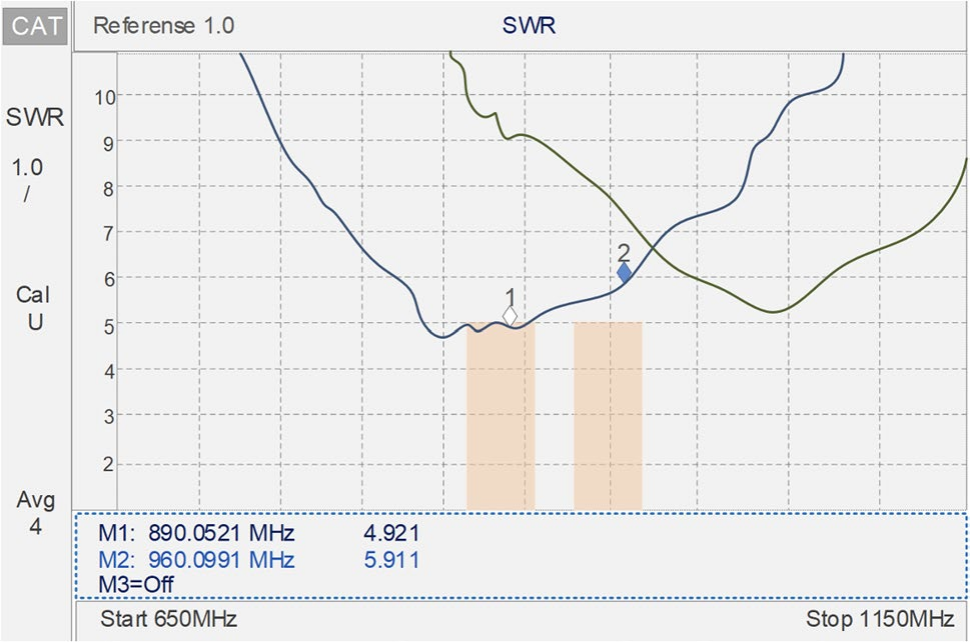
VSWR of planar F-antenna No. 8 (left trace) and antenna No. 10 (right trace) in a 2 mm thick ABS plastic enclosure.

Simultaneously, some of the examined antenna samples demonstrated extremely unsatisfactory electrical characteristics, making their use in navigation seals infeasible. This is clearly illustrated in the corresponding plots (Fig. 4), where the VSWR values exceed the acceptable limits at all reference points, and the shape of the curves indicates poor impedance matching with the radio module and high energy losses.

These antennas proved to be the least suitable for operation with plastic enclosures, even in a relatively “spacious” layout. Therefore, experiments with them in other en-closures were not performed. In addition, it should be noted that the performance of surface-mounted patch antennas within a navigation seal is largely determined by the influence of dielectric and metallic structural elements. Specifically, the intrinsic dielectric permittivity (*\varepsilon*) of the enclosure plastic alters the antenna parameters by shifting the resonant frequencies and changing the input impedance. If this factor is not considered, the antenna may move out of the operating band and lose proper impedance matching. Similarly, nearby metallic elements modify the antenna characteristics, affecting both the frequency band and the VSWR values. Therefore, the VSWR measurement at the incoming inspection stage is a critical tool for the early identification of low-quality samples: high VSWR values indicate poor matching with the radio module, leading to reduced receiver sensitivity and increased transmitter power consumption under weak network coverage conditions.

## Results

### Design and functional features of the developed navigation seal

As part of an ongoing study, a step-by-step development of a proprietary navigation seal prototype compliant with the requirements of^1^ was conducted. Currently, comprehensive laboratory testing is being conducted on individual device modules to verify their functionality and compliance with the specified characteristics. The structural design and layout were developed using the SolidWorks computer-aided 3D modeling system, which makes it possible to consider the requirements for mechanical strength, dimensions, manufacturability, and rational component placement at the design stage.

First, it was necessary to determine the approach and strategy for using an external patch antenna for transmitting short messages in the GSM 900 band. When integrating the antenna into the controller board, certain layout features of the navigation seal must be taken into account. The antenna must meet the following criteria:

- Simplicity of design;
- Relatively small mass and dimensional characteristics;
- Low production cost;
- High repeatability of characteristics.

The orientation of the radiating elements should form a polarization close to vertical in accordance with the recommendations for GSM 900 networks and should not coincide with elongated mechanical elements. However, the orientation of the controller printed circuit board is determined by the device design and the rather dense arrangement of the internal units of the navigation seal. In order to maintain acceptable antenna efficiency, it is desirable to place it as far as possible from metallic structural elements. Therefore, we selected an antenna that is not integrated with the controller printed circuit board.

The antenna is attached to the plastic housing of the navigation seal, and its parameters change due to the dielectric permittivity of the plastic material (*\varepsilon*). This effect was clear from the beginning, whereas the influence of numerous metallic structural elements was difficult to predict. The antenna was designed specifically for the developed device using simplified idealized models, with the intention of refining the parameters during practical measurements and experimental testing^29–31^.

**Fig. 5:**
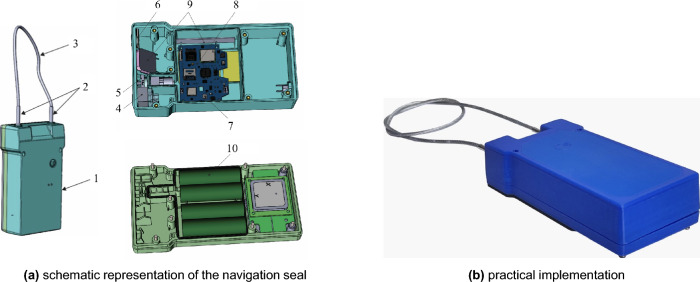
Developed navigation seal.

Figure 5 shows the external appearance of the experimental prototype of the developed navigation system. The device enclosure is made of durable and wear-resistant plastic, providing protection against moisture and dust, in accordance with the IP67 rating.

**Fig. 6:**
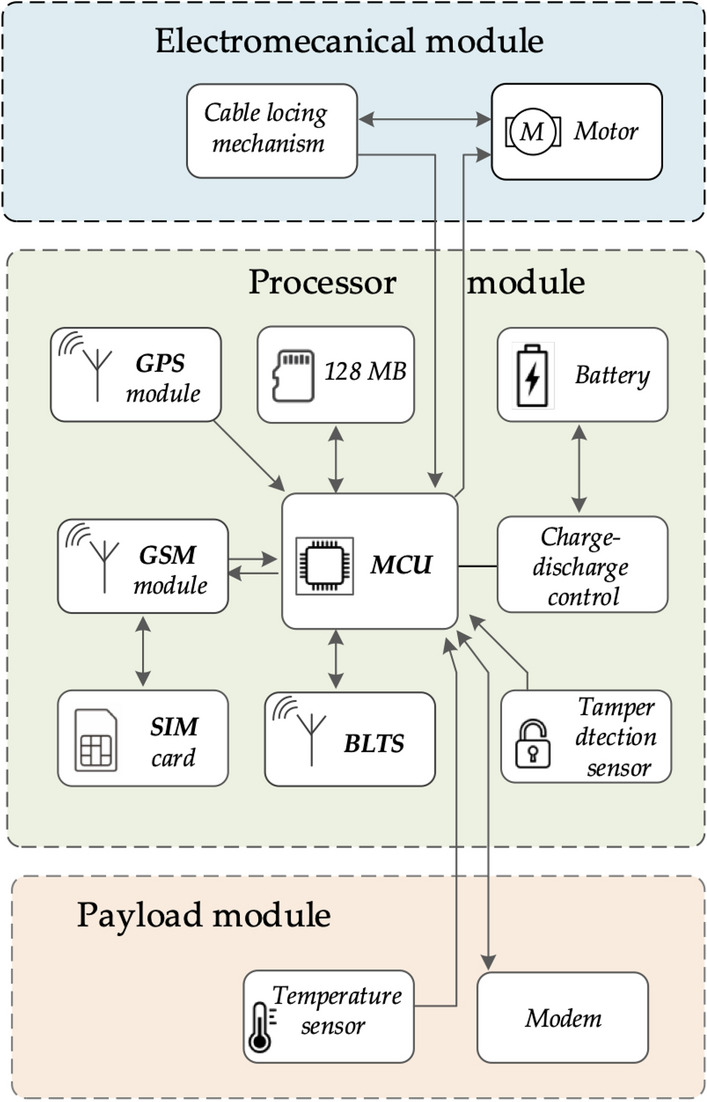
Block diagram of the developed modular navigation seal.

Figure 5 presents the design of the developed navigation seal, which includes several functional elements housed within an impact-resistant and moisture-protected enclosure (1). A key component of the device is the electronic unit (8), which performs multiple functions by integrating a combined control and communication module with a built-in antenna (9). This unit not only monitors the internal processes within the seal but also enables data exchange with an external information system. The mechanical part of the seal consists of a sealing element (2) connected to cable (3). This cable serves as the primary component to ensure the physical integrity of the seal. The cable fastening system includes a mechanism (5) with a servomotor that allows the cable to be securely locked or released depending on the received control commands. In addition, the proposed system provides a high level of protection against unauthorized tampering attempts. The enclosure condition is monitored by a sensor (7), which responds to any attempts to open it. This function is critically important for preventing unauthorized access to the contents of seals. The combined module (8), which integrates microprogram control and data processing, plays a central role in the operation of the seal and ensures its real-time functionality. The power for the seal components is supplied by a battery (10) located inside the enclosure. It provides long-term autonomous operation between external recharging cycles, ensuring the reliable and prolonged use of the seal under conditions where access to power sources is limited or unavailable. Thus, the developed navigation seal shown in Fig. 5 is a multifunctional device that provides a high level of security during the transportation of valuable cargo owing to its well-engineered design. To ensure operational reliability and ease of maintenance, the navigation seal was designed according to the modular architecture principle. Figure 6 shows a block diagram of the developed navigation seal. It highlights an electromechanical module (responsible for monitoring the integrity of the sealing element and providing physical protection), processing module (performing navigation and diagnostic data processing as well as communication control, including the GSM antenna), payload module, and additional sensors of the device. This approach makes it possible to extend the system’s functionality by integrating additional communication channels or sensors (e.g., vibration, temperature, and open/close detection).

**Fig. 7:**
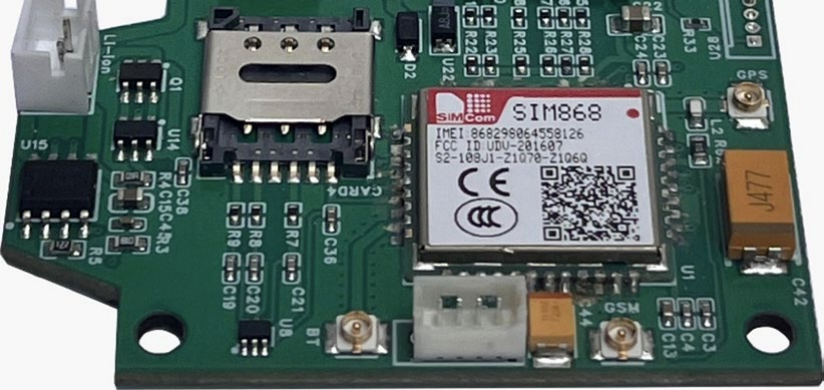
GSM SIM868 module with an external antenna and control board.

Thus, the modular structure ensures high energy efficiency, as individual device units can be switched off or placed in sleep mode depending on the tasks and system state. This significantly extended the autonomous operating time of the navigation seal, as confirmed by the experimental results from laboratory tests. The GSM SIM868 module (Fig. 7) was used as the main component for organizing wireless data transmission in the developed system.

This module is mounted directly on the main board of the device, and the external antenna is connected via a u.fl connector. In modern mobile devices, compact F-type antennas are widely used, which require the use of the PCB ground plane as a counterpoise. To avoid dependence on the PCB ground plane, we employed a meandered dipole design on an FR-4 substrate with a cable connection and a microstrip matching element^32,33^. The dipole structure is symmetrical and does not require a ground plane.

Unlike an F-type antenna, feeding a dipole through a cable requires a balancing arrangement. To avoid complicating the matching elements and to reduce the antenna effect of the cable, this issue was addressed by routing the cable along one of the dipole arms (see Fig. 1).

The module architecture includes a microprocessor that controls the radio frequency and network functions, as well as indicator LEDs for the visual monitoring of the operation and connection status. This solution ensures the reliable operation of the navigation seal under intensive operating conditions and simplifies system integration into various logistics scenarios.

### Results of laboratory testing of antennas

The quality and reliability of data transmission in wireless systems largely depend on the characteristics of the antennas used. Therefore, the next important stage of this study involved the selection, analysis, and experimental evaluation of various antenna solutions. Under conditions of limited internal enclosure volume, the presence of metallic components, and the need to maintain sealing, selecting an optimal antenna is critical to ensuring stable device operation. Within the scope of this study, a series of laboratory and field tests of antennas was conducted under conditions as close as possible to the real operating scenarios of the navigation seal.

The transmission coefficient at a frequency of 890 MHz is approximately 0.8 dB relative to a reference *λ /4* antenna.

However, we do not see any practical value in presenting the radiation pattern of the “Zmeyka” antenna or F-type antennas in free space. Under real operating conditions, navigation seals will spend most of their time between metal containers, and multiple reflections will transform the actual radiation pattern into something resembling a fan. Under such conditions, it is much more useful to study the overall performance of the antenna and the radio module under conditions close to real operation. This was the objective of this work.

**Fig. 8:**
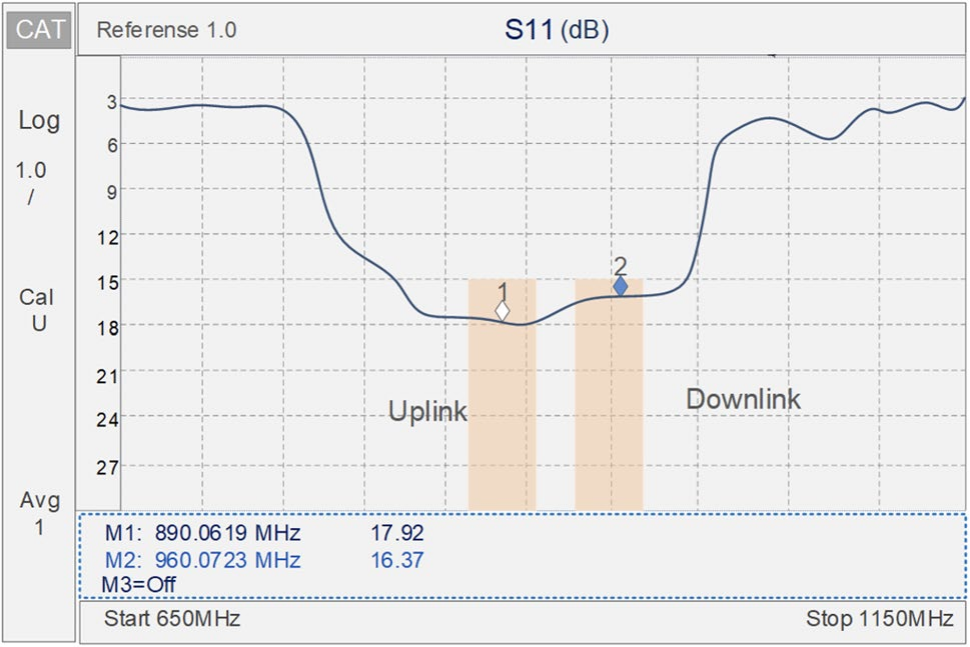
Return loss ($S_{11}$) of the “Zmeyka” antenna in a prototype housing made of ABS plastic with a thickness of 2 mm.

The following section presents the return loss (S11) of the “Zmeyka” antenna in a prototype housing made of ABS plastic with a thickness of 2 mm (Figure 8).

The return loss of the “Zmeyka” antenna was about 18 dB at a frequency of 890 MHz and 16.4 dB at a frequency of 960 MHz. This is a good result considering the influence of the dielectric permittivity of the plastic and the nearby metallic structural elements.

**Fig. 9:**
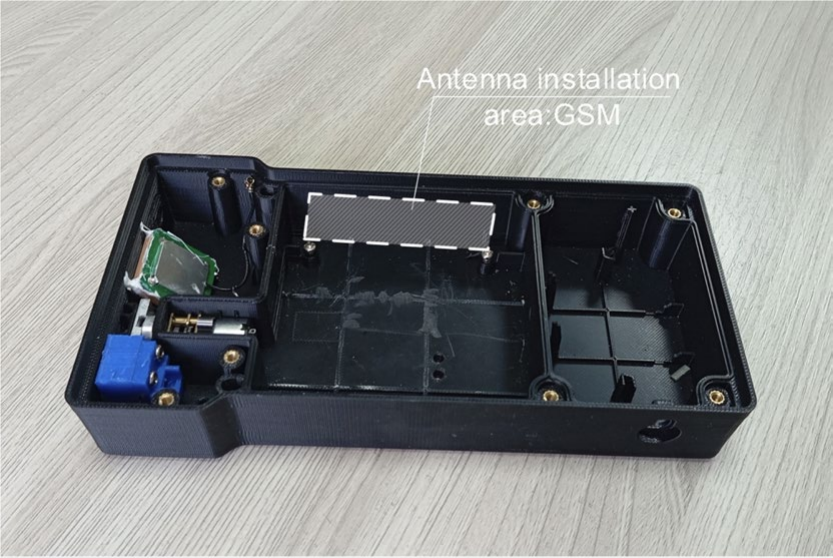
Photograph of the new navigation seal enclosure made of ABS plastic.

The VSWR is an important radio frequency characteristic of an antenna that indicates the degree of matching between the antenna and the transmitting path of the device. A high VSWR value indicates significant power losses owing to reflections in the antenna–transceiver transmission line, leading to reduced transmission efficiency and a lower output signal level of the radio module. This can degrade the signal-to-noise ratio, reduce data transmission reliability, and increase the likelihood of connection loss, particularly in areas with unstable mobile network coverage. Consequently, minimizing the VSWR contributes to improved energy efficiency, operational stability, and overall reliability of the electronic navigation seal under real operating conditions. As the project progressed, the device layout became increasingly compact. To ensure reliable operation under real operating conditions, including low temperatures, mechanical shocks, and exposure to road chemicals, a housing made of a stronger material with thicker walls is required. The battery capacity was also increased, and an additional module for accommodating a satellite radio modem was installed inside the enclosure, which further reduced free space around the antenna. Thus, considering the design changes and denser layout, the updated enclosure (Fig. 9) required reassessment of the available area for antenna installation. This became particularly important after the integration of the satellite radio module, increase in battery capacity, and thickening of the enclosure walls.

The dashed line indicates the area of GSM antenna placement. The photograph shows that the free space around the intended antenna location is significantly restricted. This necessitates the additional optimization of the shape and placement of the antenna element, considering electromagnetic compatibility and the influence of neighboring components. In this study, various types of plastics were tested, including ABS, PLA, PETG, and reinforced PA12+GF. The objective of these tests was to select a material that provides an optimal balance between 3D printing manufacturability, resistance to loads during transportation and suitability for subsequent thermoforming in serial production. The operating temperature range of PLA plastic is significantly lower than that of ABS plastic. Due to its high hygroscopicity, the use of this polymer in cold weather poses difficulties, and its upper temperature limit is approximately 60–70 °C. PLA exhibits high stiffness, is resistant to ultraviolet radiation, and has low flammability. PLA is a dielectric material with a relative permittivity of approximately 3.2. PETG is a polymer based on polyethylene terephthalate modified with glycol. It is characterized by its high strength and impact resistance. Most manufacturers specify an operating temperature range of up to +75 °C. PETG is resistant to most chemical agents and is non-hygroscopic. The dielectric constant of the material is approximately 2.6. In terms of structural parameters, PA12+GF plastic was the most suitable choice. It meets the operating conditions of the navigation seal, withstands exposure to chemical road reagents and petroleum products, impact loads from stones and accidental drops, and endures temperature fluctuations typical of a sharply continental climate. PA12+GF (polyamide 12 + Glass Fiber) is a composite material and a type of nylon reinforced with glass fibers (up to 30 After manufacturing working prototypes of navigation seal enclosures from various types of plastics, a selection of antennas previously measured in terms of VSWR (Table 2) was performed based on the criteria of excellent and good VSWR performance, as well as the feasibility of their placement within the fabricated enclosures. The maximum allowable dimensions of the antenna installation area inside the electronic seal enclosure were 70 mm × 20 mm.

**Table 2:** Polyamide F-type antennas compliant with the dimensions of the new enclosures.

Antenna no.	VSWR (890 MHz—uplink)	VSWR (960 MHz—downlink)	Average VSWR	Assessment
0 “Snake” (reference)	1.13	1.27	1.20	5
2	1.70	1.68	1.69	5
6	1.66	2.00	1.83	5
24	2.51	2.70	2.60	4
25	1.63	1.52	1.58	5
26	2.22	2.86	2.54	4

**Fig. 10:**
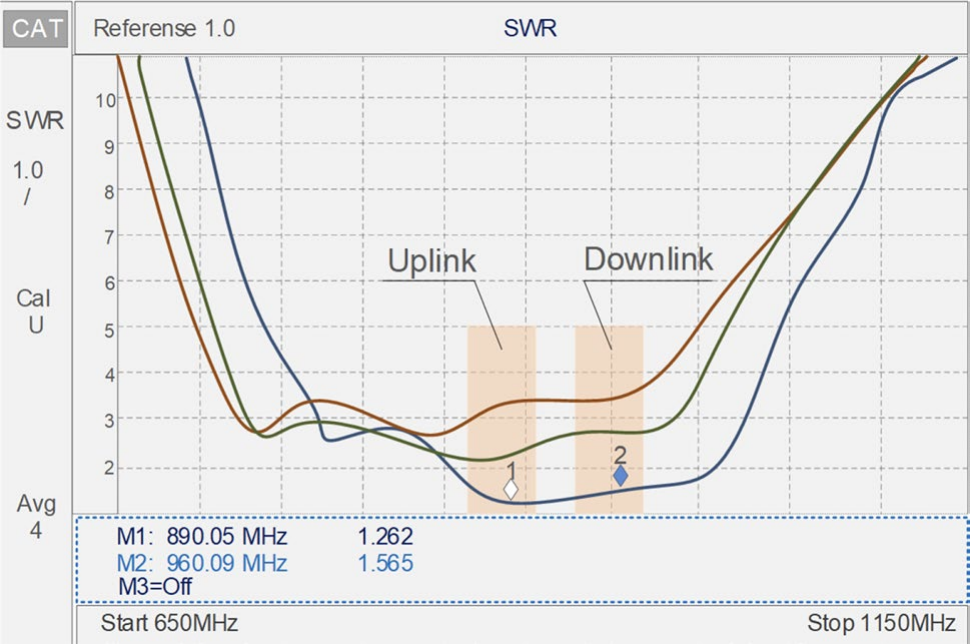
Degradation of the SWR coefficient of the “Snake” antenna in different enclosures.

The “Snake” antenna and five commercial antennas met the dimensional constraints of the new enclosure manufactured using 3D printing. The VSWR evaluation values were obtained from preliminary studies conducted on an enclosure mock-up ( Table 2). For mass production, the PA12+GF material is planned to be selected. This nylon, which offers high strength and resistance to temperature fluctuations, has a dielectric permittivity in the range of 3.5–4.5, which exceeds that of ABS. This implies that an antenna placed inside an enclosure made of this material will significantly alter its original characteristics. Consequently, the initial “comfortable” operating conditions for the GSM antenna deteriorated. We found that the meander-type “Snake” antenna, which demonstrated the best performance in an ABS plastic enclosure with a thickness of 2 mm and a more “spacious” layout (lower trace in Fig. 10), exhibited degraded SWR parameters when installed in an enclosure made of PA12+GF plastic with a thickness of 3.5 mm. In this case, the SWR increased to 2.2 (uplink) and 2.7 (downlink), and the resonant tuning band shifted toward lower frequencies (to the left), as shown by the green trace in Fig. 10. The installation of battery packs inside the enclosure in close proximity to the antenna (with a minimal gap) further worsened the SWR to 3.5 and caused an additional downward shift of the resonant tuning band. Figure 10 shows the degradation of the SWR coefficient resulting from the new navigation seal enclosure layout.

**Fig. 11:**
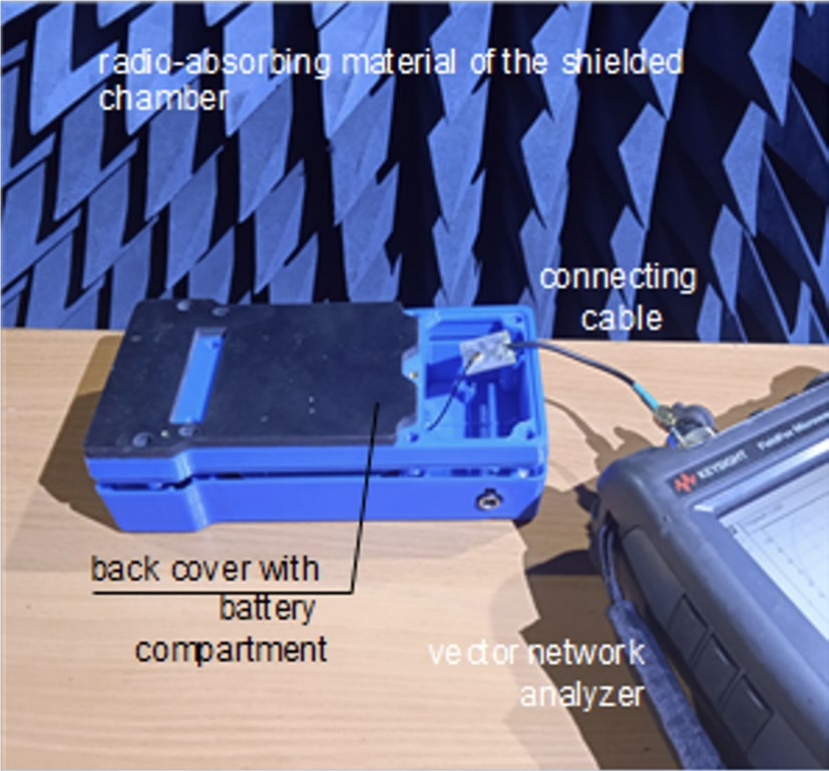
Photograph of SWR measurement of the “Snake” antenna in a PETJ plastic enclosure with the rear cover closed, inside the anechoic chamber.

Consequently, it is evident that a dense layout and the presence of batteries negatively impact antenna impedance matching. That is, the degradation of the SWR coefficient in both frequency bands occurs not only because of changes in the dielectric permittivity of the plastic, but also to the same extent because of the close proximity of the battery when the navigation seal cover is closed (Fig. 11).

**Fig. 12:**
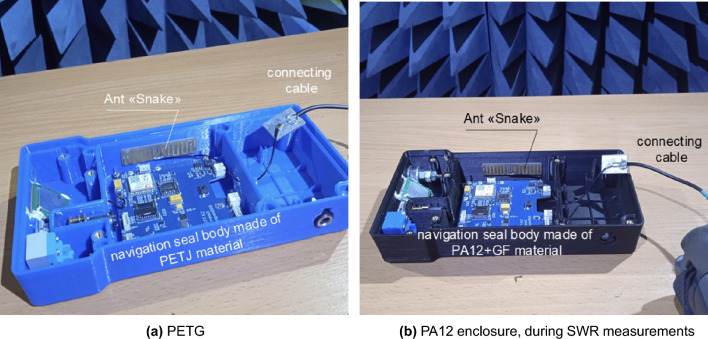
Placement of the “Snake” antenna in plastic enclosures.

To understand how the enclosure material affects the antenna performance, two experimental navigation seal prototypes with identical internal layouts were assembled. One was made of PETG, and the other was made of reinforced PA12+GF. The position of the “Snake” antenna, cable routing, and placement of components remained unchanged. A visual comparison is presented in Fig. 12.

**Fig. 13:**
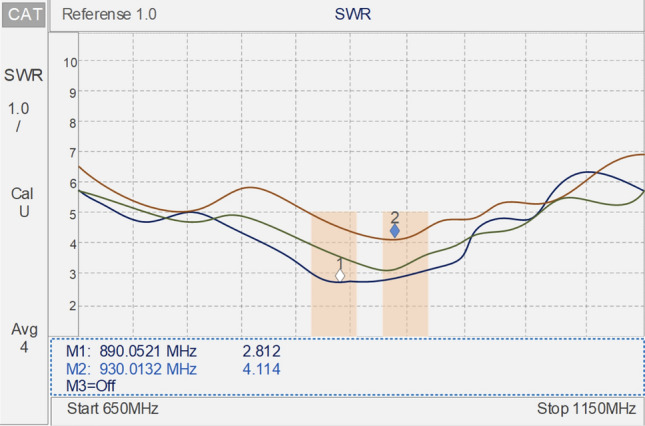
SWR coefficient of planar polyamide F-antenna No. 2 in different enclosures.

Theoretically, in an approximate form, an increase in the effective dielectric permittivity of the antenna substrate by a factor of *\varepsilon* due to the enclosure plastic should shift the antenna tuning frequency by a factor of . The transition from ABS material with an average permittivity of approximately 3 to PA12+GF with an average permittivity of approximately 4 practically reduced the cutoff frequency of the “Snake” antenna by approximately 50 MHz. This is clearly visible on the right slope of the SWR curve shown in Fig. 4. Subsequently, similar SWR measurements were performed on commercial antenna samples installed in different seal enclosures. It was assumed that commercial antennas with different substrates, configurations, and thicknesses might exhibit less distortion of their parameters when integrated into the enclosures. The following plots show the changes in impedance matching and frequency detuning in the different enclosures (Fig. 13).

**Fig. 14:**
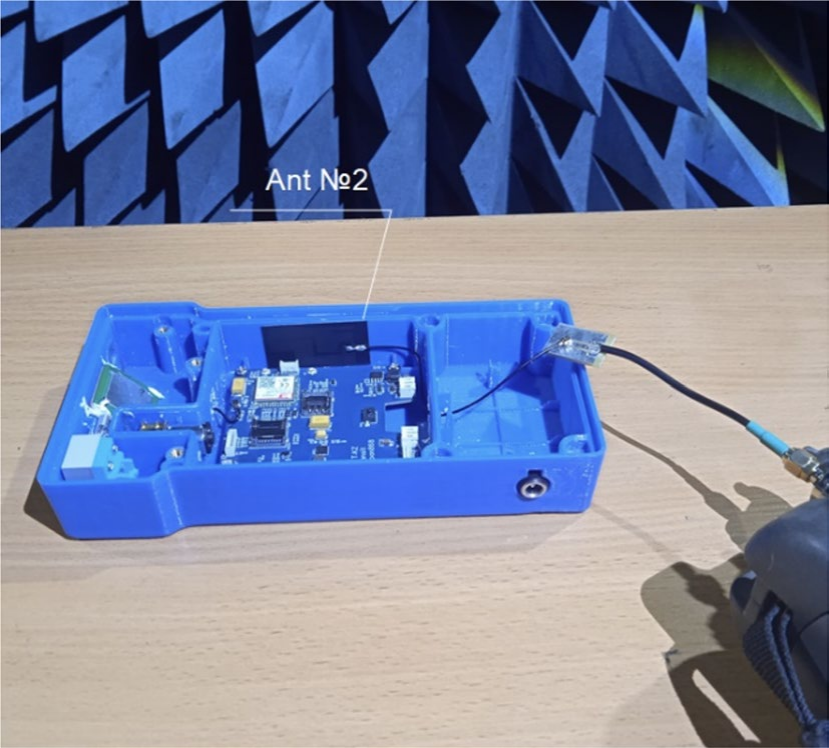
Placement of antenna No. 2 in the PETG enclosure during SWR measurements.

The graph illustrates how the SWR of the planar antenna changes depending on the enclosure material. The lower trace corresponds to the configuration with an ABS plastic enclosure of 3 mm thickness and dense internal layout. The middle trace represents an enclosure made of PETG with a thickness of 3.5 mm. The upper trace corresponds to an enclosure made of reinforced PA12+GF with the same thickness. The measurements showed that commercial antennas are no less sensitive to the surrounding material than the “Snake” antenna, particularly in the case of F-type antennas. When polyamide (PA12+GF) was used, a shift in the resonant frequency toward higher frequencies was observed. The markers on the graph are placed at the points of minimum SWR and clearly show the degradation of impedance matching and the shift of the antenna tuning band to the right, that is, toward higher frequencies. For clarity, a photograph (Fig. 12) is also provided, showing the placement of antenna No. 2 (F-type model) in a PETG enclosure, during the measurements. A visual representation of the placement of antenna No. 2 in the PETG enclosure without the cover is shown in Fig. 14.

**Fig. 15:**
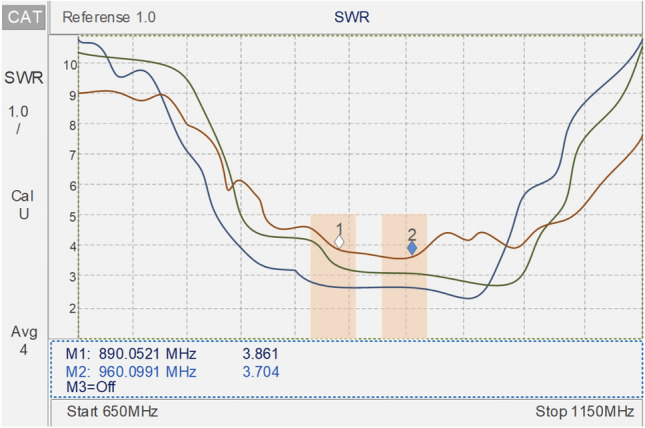
SWR coefficient of planar F-antenna No. 25 in different enclosures.

A similar behavior was observed for F-antenna No. 25 (Fig. 15). As an example, VSWR curves are presented for three enclosures with dense internal layouts.

The lower trace corresponds to the new enclosure made of ABS plastic with a thickness of 3.5 mm and a dense internal layout of the components. The middle trace represents an enclosure made of PETG plastic with a thickness of 3.5 mm. The upper trace corresponds to an enclosure made of PA12+GF plastic with a thickness of 3.5 mm (Table 3).

**Table 3:** Table of VSWR measurements of selected commercial antennas in different enclosures.

Antenna GSM no.	Average SWR		
	ABS enclosure	PEDJ enclosure	PA 12 enclosure
2	2.90	3.30	4.20
6	2.97	3.34	4.31
24	3.20	3.48	4.63
25	2.55	3.13	3.76
26	3.14	3.41	4.60

In practice, the measurement results are at the boundary between satisfactory and unsatisfactory performance. Therefore, the use of such antennas with these types of enclosures is not recommended.

### Experimental evaluation of GSM antenna reception performance based on CSQ/RSSI metrics

**Fig. 16:**
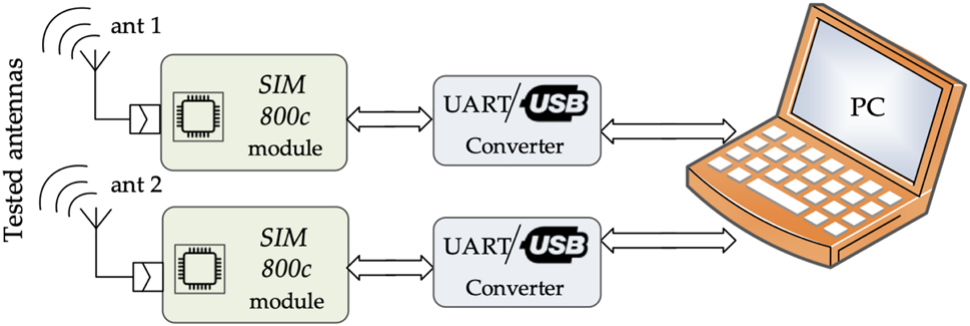
Block diagram of the GSM antenna tester.

The next step of the comprehensive evaluation was to determine the actual effectiveness of the “antenna–radio module” system using field CSQ/RSSI measurements under conditions close to real operation. For a comprehensive assessment of the antenna performance within the seal, the SIM800C radio module was used. After network registration in an area with weak coverage, the signal quality parameter AT+CSQ was measured. The CSQ value is a standard digital indicator that reflects the received signal level. Higher CSQ values indicate better reception quality and, consequently, better antenna performance under real operating conditions. The CSQ is proportional to the RSSI level in dB and enables an objective comparison of the effectiveness of different antennas in areas with minimal operator signal strength. In addition, the maximum allowable dimensions for antenna installation inside the electronic seal enclosure were considered: the length did not exceed 70 mm, and the width did not exceed 20 mm. As part of the experimental studies, a specialized software tool named “GSM Antenna Tester” was developed to automate and standardize the testing procedure of GSM antennas and modules, such as the SIM868. This software product was implemented in Python using the pyserial and PyQt5 libraries, providing a user-friendly graphical interface and reliable operation with the UART interface via a USB–UART adapter. A structural diagram of the tester is shown in Fig. 16.

**Fig. 17:**
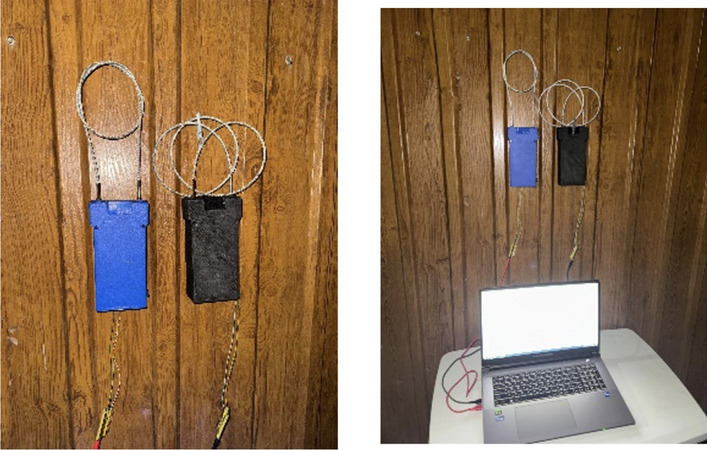
Field measurements of the received mobile network signal quality (RSSI) of the SIM800C radio module integrated into the navigation seal, with registration of the AT+CSQ parameter on a PC. The blue enclosure is made of PETG, and the black enclosure is made of PA12+GF.

For clarity in the measurement system operation, Fig. 16 presents a block diagram of the GSM antenna tester. The scheme includes two SIM800C radio modules, each connected to the tested antenna. Data transmission was carried out via UART–USB converters to a personal computer, where CSQ parameters were recorded using specialized software developed by specialists at the «Institute of space technique and technology». This configuration enabled the simultaneous testing of the two antennas and an objective comparison of their characteristics under identical conditions. Figure 17 shows an external view of the measurement test bench used for the field bench measurements.

**Fig. 18:**
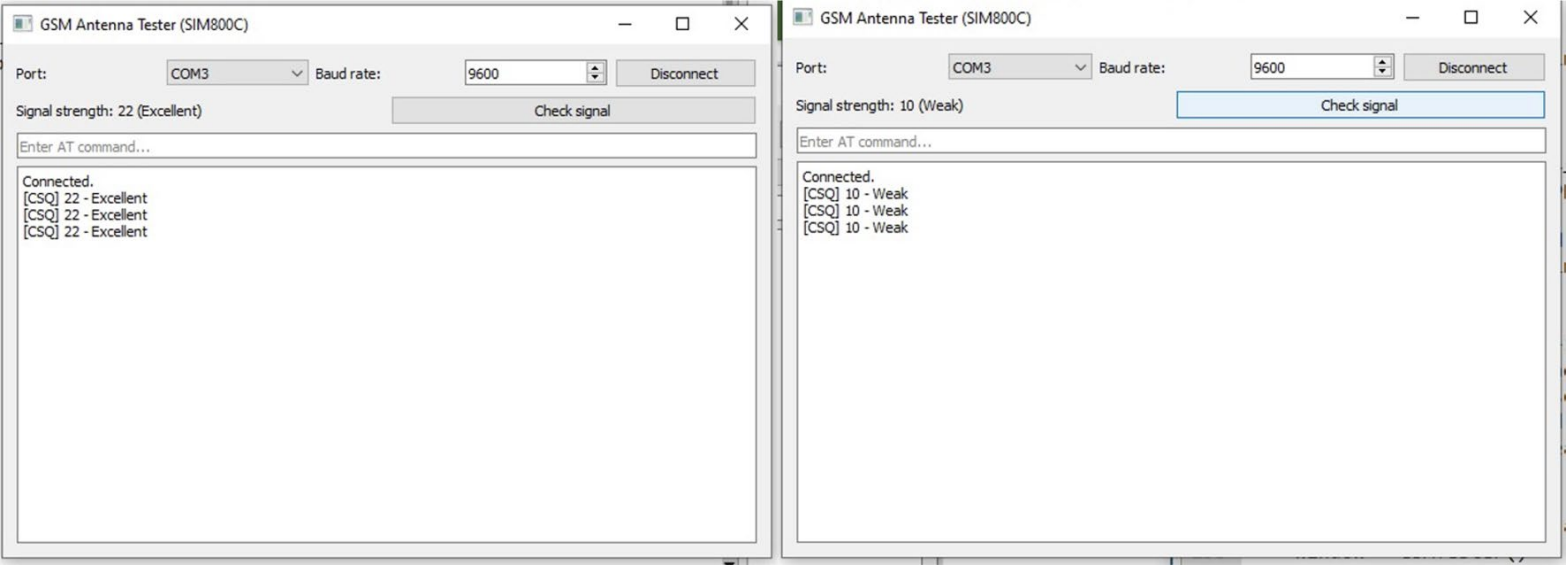
Examples of the graphical user interface of the “GSM Antenna Tester” software.

To bring the field bench measurements closer to the real operating conditions, a metallic underlying surface was used to simulate the wall of a container. The software allows the automatic detection and selection of available COM ports, configuration of the communication baud rate (default 9600 baud), and connection and disconnection of the selected GSM module. An important functional feature is signal-level polling using the standard AT command AT+CSQ. All acquired data were logged within the application and used for an objective comparison of the operational characteristics of the different antennas. Figure 18 presents the software interface that enables the control of the measurement process and real-time monitoring of the parameters of the tested antenna.

As part of the tests, various types of GSM antennas connected to the SIM868 module via a u. fl connector were evaluated. For each antenna, a series of RSSI values was recorded, which reflected the signal level under specific experimental conditions. The interpretation of the digital signal quality indicator CSQ was carried out in accordance with 3GPP TS 27.007 V17.2.0, which defines the correspondence between CSQ [0; 31] and RSSI levelsfrom $-113 \le \textrm{RSSI} \le -51$ dBm with a uniform step of 2 dB. Because the levels were uniformly quantized, a linear relationship was used for the calculation:

1 $$\begin{aligned} \textrm{RSSI}_{\textrm{dBm}}(\textrm{CSQ}) = {\left\{ \begin{array}{ll} \le -113, & \textrm{CSQ} = 0, \\ -113 + 2 \cdot \textrm{CSQ}, & 1 \le \textrm{CSQ} \le 30, \\ \ge -51, & \textrm{CSQ} = 31, \\ \text {undefined}, & \textrm{CSQ} = 99. \end{array}\right. } \end{aligned}$$

For the boundary codes of the CSQ scale, saturation to *-113* and *-51* dBm was applied, since according to the 3GPP TS 27.007 V17.2.0 (2021-06) specification, the extreme levels are defined by inequalities (*łe -113* and *\ge -51* dBm), and exact values outside the scale are not specified^34^. CSQ = 99 values were interpreted as “signal not detected” and excluded from the RSSI calculations as missing measurements. During the analysis, all obtained digital values were interpreted in accordance with ^2^, where a strict power grading was defined for each value of the “RSSI” parameter, ranging from 0 (no higher than *-113* dBm) to 31 (*-51* dBm and above). In addition, signal quality categorization for analyzing antenna effectiveness was performed in accordance with ^2^: RSSI values from *-113* to *-101* dBm (CSQ 0–6) were classified as “Very weak” signal, from *-99* to *-91* dBm (CSQ 7–11) as “Weak,” from *-89* to *-81* dBm (CSQ 12–16) as “Good,” and values above *-81* dBm (CSQ 17–31) as “Excellent” signal level. A CSQ value of 99 indicates that the signal cannot be determined. Consequently, this approach provides an objective, standardized interpretation of the communication quality for each tested antenna and enables a direct comparison of the operational characteristics in physical measurement units ^2^. The results of testing various antenna types, including the CSQ values, calculated RSSI, signal quality category, and application recommendations, are presented in Table 4.

**Table 4:** Comparative characteristics of GSM antennas by signal level.

Antenna No.	ABS enclosure	PETG enclosure	PA12+GF enclosure	Signal category
	CSQ / RSSI, dBm	CSQ / RSSI, dBm	CSQ / RSSI, dBm	
1	8 / − 96	8 / − 97	7 / − 98	Weak
2	8 / − 96	8 / − 97	7 / − 98	Weak
3	7 / − 97	7 / − 98	7 / − 99	Weak
4	12 / − 88	12 / − 89	11 / − 90	Weak
5	8 / − 96	8 / − 97	7 / − 98	Weak

When all the antennas were installed under dense internal layout conditions, their performance deteriorated noticeably. Moreover, the enclosure made of PA12+GF exhibited the worst results. Data analysis showed that all selected antennas exhibited signal levels corresponding to the “Weak” category. Therefore, the use of these antennas in the developed navigation seal with this configuration is not recommended.

### Measurement of antenna radiation patterns

**Fig. 19:**
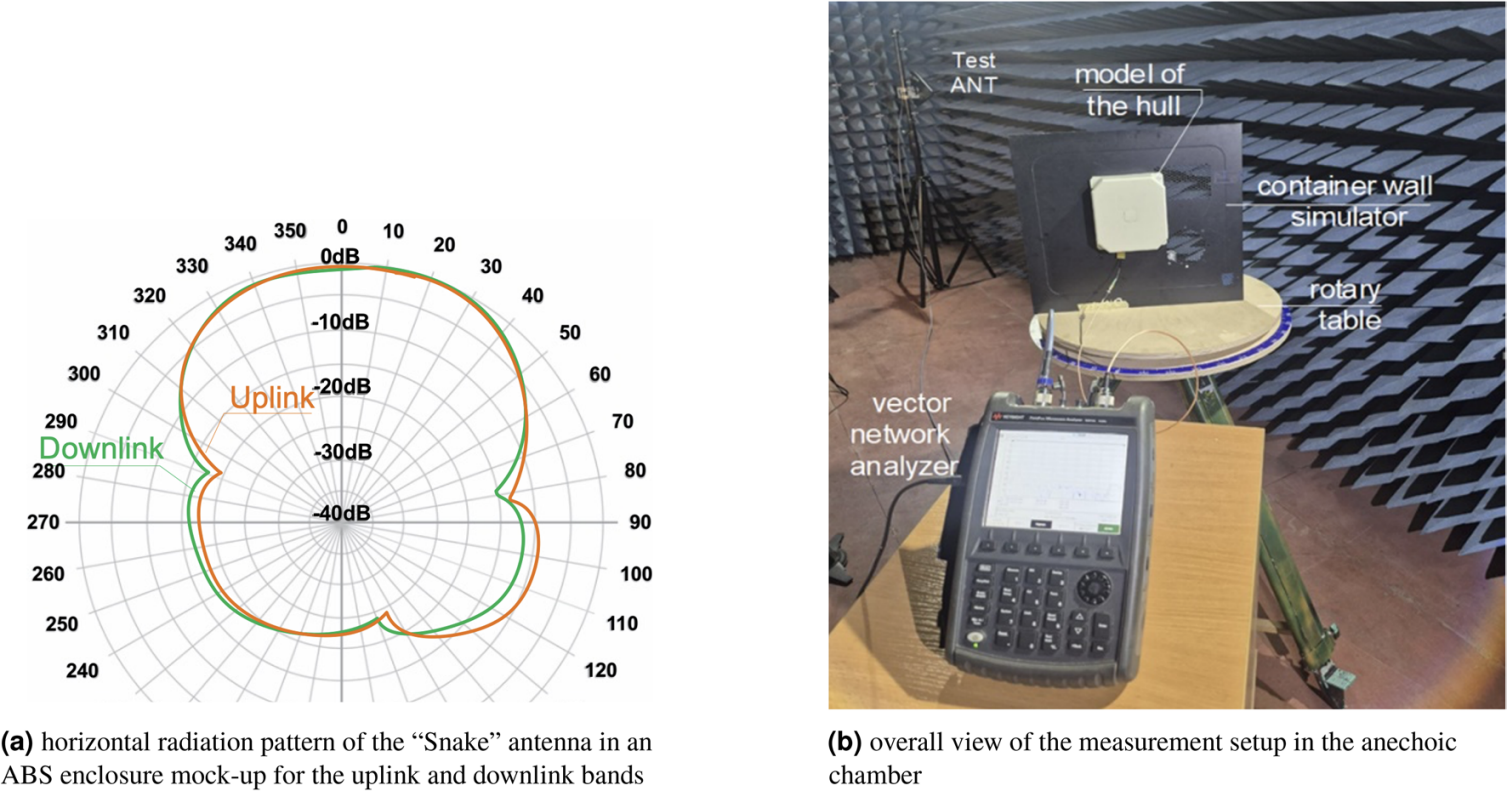
Radiation pattern of the GSM antenna in the ABS housing prototype and the measurement setup: (**a**) Horizontal radiation pattern of the “Zmeyka” antenna for the Uplink (890 MHz) and Downlink (960 MHz) bands; (**b**) General view of the measurement setup in the anechoic chamber showing the position of the ABS housing prototype (model of the housing) and the rotary table at the moment of measuring the back lobes of the radiation pattern.

Within the scope of this study, it was also necessary to examine the radiation pattern (RP) characteristics of a GSM antenna installed inside a navigation seal mounted on a metallic wall of a transport container. There are several methods for measuring radiation patterns, such as using a drone flight with a field strength indicator. In our case, a shielded anechoic chamber (SAC) at the «Institute of space technique and technology» was available, with a measurement distance of approximately 3 m. The partial simulation of a metallic container was achieved using a personal computer side panel with dimensions of 410 × 450 mm. This is, of course, an insufficient and non-ideal reflector given the GSM-900 wavelength of approximately $\lambda \approx 0.33$ m, and therefore only an approximate radiation pattern can be obtained for this case. Although the radiation pattern is an important parameter, we proceeded with the assumption that its practical value for navigation seals is ultimately limited. In real operations, the device is often located between metallic containers, leading to multiple reflections and significant distortion of the actual radiation pattern. To evaluate operational efficiency, the analysis of the integrated performance of the “antenna–radio module” system using the CSQ/RSSI metric is significantly more informative, which was implemented in the present study. For antennas exhibiting pronounced directional radiation patterns, the following conclusions drawn by the authors of studies on the influence of plastic, ceramic, and other coatings, radomes, and similar structures on antenna parameters are applicable. When the dielectric material of the enclosure is located directly in the antenna aperture region, complex electrodynamic interactions occur between the antenna field and dielectric, leading to multiple reflections of surface waves. These surface waves, which are re-radiated owing to diffraction at the edges, alter the antenna radiation pattern. As a result of radiation in such a complex environment, antenna mismatch occurs, radiation efficiency decreases, and the shape of the radiation pattern is significantly distorted^35^. In our case, the radiation pattern was not highly directional; therefore, we used a simple test setup. Figure 19 shows the radiation pattern parameters of the “Snake” antenna for the ABS enclosure.

**Fig. 20:**
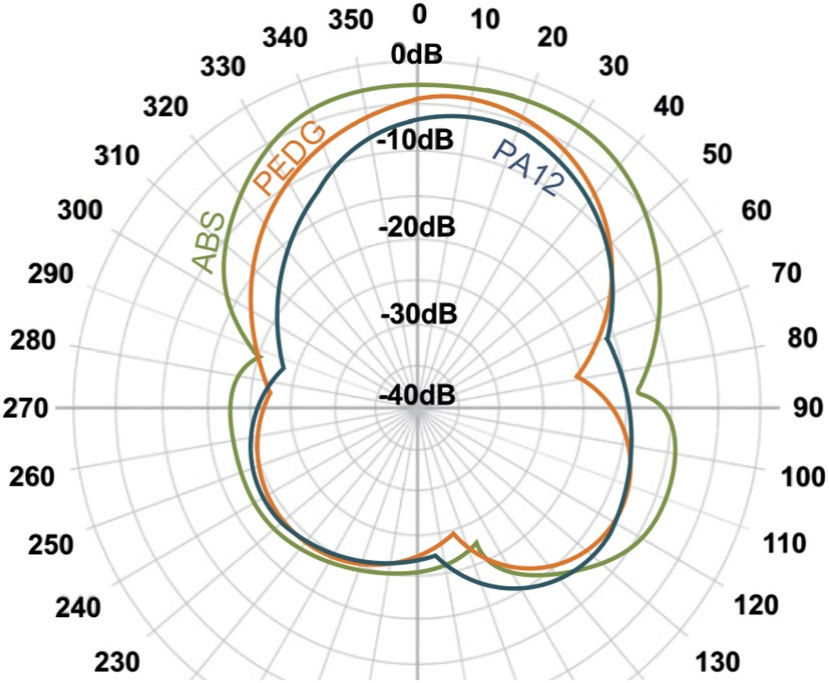
Horizontal-plane radiation patterns of antenna No. 25 in different enclosures.

**Fig. 21:**
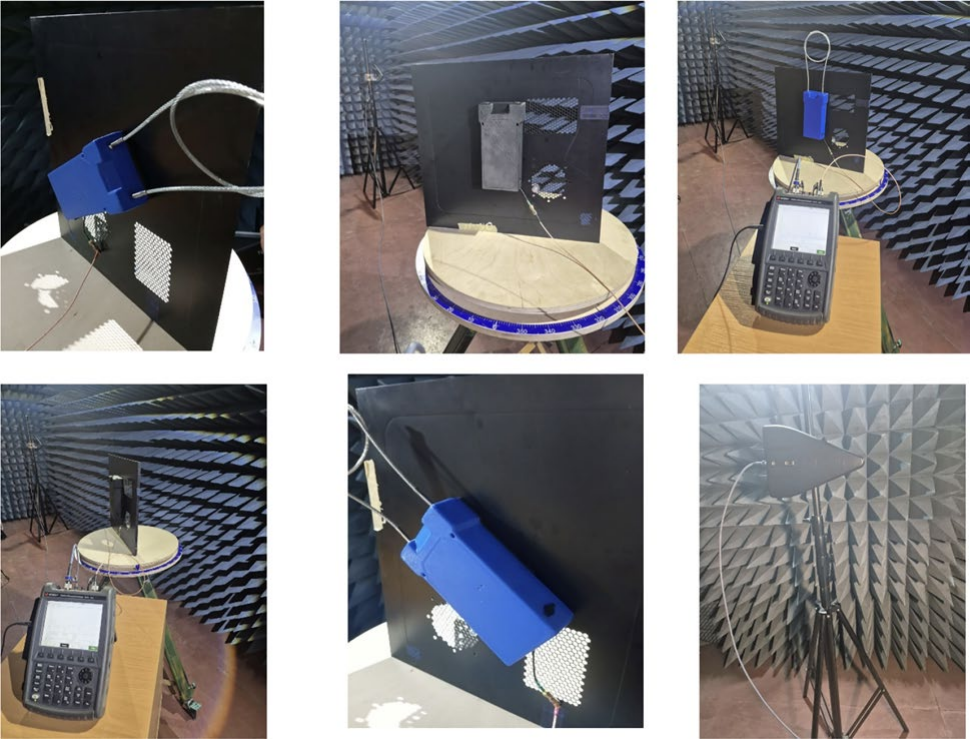
Radiation pattern measurement process for ABS, PETG, and PA12 enclosures.

The transmission coefficient of the “Snake” antenna in the most simplified enclosure mock-up was taken as zero and used as a reference for further comparative evaluation of antenna radiation patterns in different enclosures. The slight asymmetry of the radiation pattern is likely caused by the lateral placement of the GSM antenna inside the navigation seal enclosure, as well as by the “wall” simulating the container body (Fig. 3b). The radiation patterns in the uplink and downlink frequency bands almost completely coincide; therefore, only a unified radiation pattern is presented for further analysis. An example of the radiation pattern measurements in the anechoic chamber for one of the selected antennas (No. 25) installed in different enclosures is presented below (Figs. 20 and 21).

No fundamental differences in the radiation patterns were observed, except for the transmission coefficients. For the enclosure made of PA12 plastic, the degradation of the transmission coefficient was most pronounced (Fig. 21). To the right of the main lobe maximum, in the direction of 10–15°, a significant reduction in the transmission coefficient of approximately 6–7 dB was observed compared with the ABS enclosure. This is likely related not so much to losses in the plastic itself as to the VSWR mismatch of the antenna and the close proximity of metallic components of the seal, such as the controller board and the battery. The PETG enclosure exhibited a performance that was intermediate between those of the ABS and PA12enclosures. The degradation of the radiation pattern in this material occurs to a lesser extent under a similar internal configuration of seal components (battery, board, and locking mechanism). The radiation pattern shift was smaller, and the losses in the plastic were approximately 3–4 dB compared to ABS. Overall, the radiation pattern did not reveal any unexpected results, and considering the operating conditions, the obtained results were acceptable. When adapting the antenna parameters to a dense layout, the reduced transmission coefficient (*-6* to *-7* dB) should return to an acceptable level of approximately 1–2 dB.

## Discussion

The conducted measurements showed that, unlike commercial planar antennas, the in-house developed “Snake” antenna allows for further modification and tuning to specific operating conditions. The technological implementation of this antenna makes it possible to adjust the topology of the radiating and matching elements, adapting it to the enclosure geometry and internal layout of the navigation seal. However, commercial antennas do not provide this flexibility. Their design is fixed at the manufacturing stage and does not allow for modifications, making them single-use solutions intended for standard installation. Thus, the ability to adapt is the main advantage of the “Snake” antenna, which makes it a more promising option for integration into the navigation seal enclosure than the other antennas. Although the final parameters were far from ideal, the following conclusions can be drawn: First, the methodology in which antennas were initially selected based on their VSWR under laboratory conditions and then additionally evaluated using CSQ and RSSI levels in field tests proved to be effective. This makes it possible to eliminate weak options at an early stage and predict antenna performance under real operating conditions. Second, it became clear that with dense internal layouts, the presence of nearby conductive surfaces, and an unstable dielectric environment inside the enclosure, the antenna must be adapted to the specific design at the development stage. Simply using a ready-made planar GSM antenna in the device does not deliver the expected performance. Despite the rather “discouraging” overall outcome of the study, two initial assumptions were confirmed. - The method of preliminary laboratory selection of antenna samples based on VSWR, followed by verification of CSQ/RSSI parameters under real-world conditions, makes it possible to assess the future effectiveness of antennas. - Under conditions of dense navigation seal layout, strict dimensional constraints, numerous conductive elements, and increased dielectric permittivity of the enclosure, the antenna must be adapted to the specific conditions already at the design and tuning stage. It should be noted that planar antennas based on polyamide substrates are not amenable to such adaptations. They were originally designed for installation on thin plastic or glass, in other words, for “lightweight” operating conditions. Their technological implementation does not allow for modifications, which explains their low cost. Although it may be possible in laboratory conditions to manually tune an individual sample in terms of frequency range or impedance matching, this approach is impractical under mass production conditions. The complexity of developing such antennas under dense layout conditions lies in the need to account for electromagnetic compatibility rules, as well as the interaction of the power supply, control, and transmit–receive circuits with the antenna structure itself. This interaction is particularly pronounced in plastic enclosures with synthetic coatings, where the resulting integrated antenna may behave differently from what was originally expected during the design stage^36^. It should also be noted that antenna development for such devices is further complicated by the need to account for the influence of the entire circuitry, including the transmit–receive paths, power-supply lines, and control electronics. Under conditions where all these elements are enclosed within a sealed plastic housing, an initially ideal antenna may begin to behave differently. This is a typical electromagnetic compatibility issue that often manifests only after the final assembly. Therefore, against this background, the “Snake” antenna appears to be more promising for future applications. Its design allows variations in both the radiating elements and matching circuits. This makes it possible to adapt the antenna to a specific device rather than adapting the device to a fixed antenna. Such an approach offers real prospects for stable communication and reliable data transmission under field conditions, particularly in scenarios where non-adapted antennas fail to perform adequately. This is precisely what makes the “Snake” antenna a strong candidate for further integration into the navigation seal, which is required to operate not in laboratory conditions but in the real and challenging environments of transportation and logistics.

## Conclusions

This study makes an important contribution to applied radio engineering and the development of embedded, wireless devices. In practice, antenna selection is often perceived as a task that can be solved by simply choosing a solution from a catalog of ready-made products. However, the results of this study convincingly demonstrate that such an approach does not work under conditions of dense internal layouts. This is especially critical for devices with dense internal layouts, such as navigation seals intended for use in transportation and logistics chains. The results showed that the laboratory stage alone is insufficient for a reliable assessment of antenna performance in the field. Even with good impedance matching in an anechoic chamber, the antenna characteristics can degrade significantly after installation inside an enclosure, where the material properties, structural clearances, and proximity of metallic components have a strong influence. This highlights the need for a comprehensive approach that combines laboratory and field testing when designing devices in which radio-frequency parameters are of critical importance. This is especially relevant when materials with high dielectric permittivity, such as reinforced polyamide (PA12+GF), are used, or when large metallic elements are located nearby. Under these conditions, shifts in the resonant band, increases in the VSWR, and reductions in the signal strength to unacceptable levels in field tests were observed. This leads to an important practical conclusion: when designing such devices, materials, geometry, and potential electromagnetic effects must be considered from the outset, rather than attempting to adapt the antenna after development has already been completed. The proposed two-stage antenna evaluation methodology–first based on VSWR measurements in an anechoic chamber, followed by an assessment of real signal parameters (CSQ/RSSI) under field conditions–has demonstrated its effectiveness and can be adopted as an engineering method for developing compact radio frequency devices. This allows weak solutions to be eliminated at an early stage and narrows the pool of candidates for further refinement of the solution. In addition, a comparison with commercial antennas confirmed that the ability to adapt the antenna design (as in the case of the “Snake” antenna) provides a clear advantage when operating under non-standard conditions, as it enables the optimization of both the radiating elements and the matching circuits for a specific device. Thus, the results of this study have direct and practical relevance. This study establishes a scientific and applied foundation for the further refinement and deployment of domestically developed navigation seals that are resilient under real operating conditions. This is particularly important for the transport and logistics sectors, where the stability and reliability of data transmission directly affect cargo monitoring and control.

## Data Availability

All data generated and analysed within the framework of this study are included in the present publication. Kind regards, Aigul Kulakayeva
